# Differential gene expression in *Varroa jacobsoni* mites following a host shift to European honey bees (*Apis mellifera*)

**DOI:** 10.1186/s12864-016-3130-3

**Published:** 2016-11-16

**Authors:** Gladys K. Andino, Michael Gribskov, Denis L. Anderson, Jay D. Evans, Greg J. Hunt

**Affiliations:** 1 Information Technology at Purdue, Research Computing, Purdue University, West Lafayette, 47907-2114 IN USA; 2 Department of Biological Sciences, Purdue University, West Lafayette, 47907-2054 IN USA; 3 Abu Dhabi Food Control Authority, Kuwaitat Research Station, Al Ain, United Arab Emirates; 4 Bee Research Laboratory, Beltsville Agricultural Research Center - East, U.S. Department of Agriculture, Beltsville, MD 20705-0000 USA; 5 Department of Entomology, Purdue University, West Lafayette, 47907-2089 IN USA

**Keywords:** *Apis mellifera*, *Apis cerana*, Asian honey bee, European honey bee, RNA-Seq, Transcriptome, *Varroa destructor*, *Varroa jacobsoni*

## Abstract

**Background:**

*Varroa* mites are widely considered the biggest honey bee health problem worldwide. Until recently, *Varroa jacobsoni* has been found to live and reproduce only in Asian honey bee (*Apis cerana*) colonies, while *V. destructor* successfully reproduces in both *A. cerana* and *A. mellifera* colonies. However, we have identified an island population of *V. jacobsoni* that is highly destructive to *A. mellifera*, the primary species used for pollination and honey production. The ability of these populations of mites to cross the host species boundary potentially represents an enormous threat to apiculture, and is presumably due to genetic variation that exists among populations of *V. jacobsoni* that influences gene expression and reproductive status. In this work, we investigate differences in gene expression between populations of *V. jacobsoni* reproducing on *A. cerana* and those either reproducing or not capable of reproducing on *A. mellifera,* in order to gain insight into differences that allow *V. jacobsoni* to overcome its normal species tropism.

**Results:**

We sequenced and assembled a *de novo* transcriptome of *V. jacobsoni*. We also performed a differential gene expression analysis contrasting biological replicates of *V. jacobsoni* populations that differ in their ability to reproduce on *A. mellifera*. Using the edgeR, EBSeq and DESeq R packages for differential gene expression analysis, we found 287 differentially expressed genes (FDR ≤ 0.05), of which 91% were up regulated in mites reproducing on *A. mellifera*. In addition, mites found reproducing on *A. mellifera* showed substantially more variation in expression among replicates. We searched for orthologous genes in public databases and were able to associate 100 of these 287 differentially expressed genes with a functional description.

**Conclusions:**

There is differential gene expression between the two mite groups, with more variation in gene expression among mites that were able to reproduce on *A. mellifera*. A small set of genes showed reduced expression in mites on the *A. mellifera* host, including putative transcription factors and digestive tract developmental genes. The vast majority of differentially expressed genes were up-regulated in this host. This gene set showed enrichment for genes associated with mitochondrial respiratory function and apoptosis, suggesting that mites on this host may be experiencing higher stress, and may be less optimally adapted to parasitize it. Some genes involved in reproduction and oogenesis were also overexpressed, which should be further studied in regards to this host shift.

**Electronic supplementary material:**

The online version of this article (doi:10.1186/s12864-016-3130-3) contains supplementary material, which is available to authorized users.

## Background

Honey bees (*Apis mellifera* L.) are the most important insect for pollination of crops and wildflowers [1–3], but they have experienced increasing colony die-offs during the past two decades [4–6]. *Varroa destructor* is widely considered the most serious risk factor for honey bee colony mortality worldwide [7–10]. These large ectoparasitic mites are associated with a condition known as parasitic mite syndrome (PMS), or “Varroosis”. In colonies exhibiting PMS or “Varroosis”, pathogens, including brood diseases and viruses, are present at unusually high levels [11–13]. *Varroa* mites feed on the hemolymph of the larva, pupa and adults, and the open wounds caused by mite feeding can allow microorganisms to enter and weaken the host [14]; Mites themselves are vectors for viruses and perhaps other bee pathogens [13]. The V*arroa* mite’s life cycle consists of two phases, the phoretic phase, during which the adult female mite lives, feeds, and disperses on the adult bee, and the reproductive phase in which the female mite feeds and reproduces inside the sealed brood cell of the pupating honey bee [15]. After a female mite invades the brood cell, the first egg laid will develop into a haploid male, which will later mate with his sisters (unless two females invade the same cell) to give rise to the next generation. The most common Varroa-associated viral infection is deformed wing virus (DWV). The incidence of DWV is closely associated with mite infestation and colony mortality, but other bee-pathogenic viruses such as acute bee paralysis virus have also been identified as part of the “Varroosis” [15, 16]. Failure to treat infested colonies with miticides typically results in colony death within 1–3 years.

*V. destructor* was originally a parasite of the Asian honey bee, *Apis cerana*. At least 60 years ago, it made a host switch and now parasitizes several European and African races of *A. mellifera* [17]. Population studies indicate that there was a genetic bottleneck associated with the host switch to *A. mellifera* [18–20]. These studies revealed a remarkable absence of heterozygosity in the *Varroa* populations of Europe and USA collected on *A. mellifera* [21–23]. Furthermore, a study using microsatellite markers in 45 different populations of *Varroa* mites from around the world showed a relative lack of polymorphisms within each of the two *V. destructor* mitochondrial haplotypes, Japan (J) and Korea (K), that successfully infest *A. mellifera* outside of Asia. These results suggested that these two haplotypes, J and K, each correspond to a single host capture event, followed by a rapid spread, particularly by K, which has now almost spread worldwide. These haplotypes also seem to be completely reproductively isolated from each other. Two routes of invasion of *V. destructor* into the Americas, and specifically into the USA, have been proposed based on the dates and places where each haplotype was first detected [18–20]. The J haplotype first shifted from *A. cerana* to *A. mellifera* in Japan during the last century, following the introduction of *A. mellifera*. From Japan, it spread to Thailand, to Paraguay in (1971), to Brazil in 1972, and was later found in North America in 1987. The K haplotype first shifted from *A. cerana* to *A. mellifera* near Vladivostok (north of the Korean peninsula), following the introduction of *A. mellifera* from Ukraine in the 1950s. Later, it spread from eastern Russia to western Russia, to Bulgaria in 1972, to Germany in 1977, and then continued spreading around Europe and also to the U.S.

Other haplotypes of *V. destructor* as well as haplotypes of a sister species, *V. jacobsoni*, are reportedly restricted to *A. cerana* and only reproduce on drone brood in this species. All of these *Varroa* mites routinely invade sympatric non-host colonies and enter the drone and worker brood, but for unknown reasons do not produce offspring, perhaps as a result of failure to recognize host signals to initiate reproduction. Single *V. jacobsoni* female mites with dead immature offspring were found inside *A. mellifera* drone brood cells in Papua New Guinea (PNG) in 1991 and 1993, and these single events were reported in 1994 [24]. Recently, a population of *V. jacobsoni* was found reproducing on *A. mellifera* drone and worker brood, and was associated with colony mortality in PNG [25]. Evidence suggests that this host switch occurred by mites first gaining the ability to reproduce on drone brood, followed by adaptation to reproduce on worker brood. Since *V. destructor* has caused widespread losses wherever it has become established, it is important to study the *V. jacobsoni* host switch to *A. mellifera* to gain understanding of the evolutionary host shift from the Asian to the European honey bee. In addition, it is important to understand how this mite has become established, whether host-parasite signaling may be involved, and what cues may be associated with alterations in mite reproduction.

It is reasonable to expect that *Varroa* mites must change their gene expression in order to grow and reproduce in a different host species. As a first step, to understand the evolutionary host shift of *Varroa* mites to a new host, we have studied the transcriptome profile of *V. jacobsoni* reproducing on *A. mellifera* and compared it to that of *V. jacobsoni* restricted to reproducing on *A. cerana*.

## Methods

### Sample collection

A total of nine samples of *V. jacobsoni* from PNG and the Solomon Islands (Table 1) were collected from either *A. cerana* or *A. mellifera* drone brood cells during April 2010. When collected, their reproductive status (reproducing or not reproducing) was recorded. *V. jacobsoni* reproducing on *A. mellifera* were collected from Goroka, PNG, and will be referred in the rest of this paper as (Am-reproductive). Samples collected from St. Christobel Island (SC) and Ugi Island in the Solomon Islands were mites that were reproducing on *A. cerana* and will be referred in the rest of this paper as (Ac-reproductive). In addition, single adult females from *A. mellifera* colonies on SC and Ugi Island in Solomon Islands were found in brood cells but were not reproducing (mite offspring were not present) and will be referred in the rest of this paper as (Am-non-reproductive). It had been previously noted that over the previous 3 years mites on SC and Ugi Island could not reproduce on either worker or drone brood after spreading from sympatric *A. cerana*. All samples were collected in RNAlater® and stored at -80 °C until RNA extraction.

**Table 1:** Description of *V. jacobsoni* RNA samples

Bee host	Reproductive status	Collection	Collection sites	Year of sequencing
*A. cerana*	Reproducing	Drone cells	SC^a^, Solomon Islands	Apr 2012 (HiScanSQ)
*A. cerana*	Reproducing	Drone cells	Ugi, Solomon Islands	Jan 2013 (Hiseq2000)
*A. cerana*	Reproducing	Drone cells	Guadalcanal, Solomon Islands	Jan 2013 (Hiseq2000)
*A. mellifera* ^b^	Non-reproducing	Drone and worker cells	SC and Ugi (Solomon Islands)	Apr 2012 (HiScanSQ)
*A. mellifera*	Reproducing	Drone cells	Goroka, Papua New Guinea	Apr 2012 (HiScanSQ)
*A. mellifera*	Reproducing	Drone cells	Goroka, Papua New Guinea	Jan 2013 (Hiseq2000)
*A. mellifera*	Reproducing	Drone cells	Goroka, Papua New Guinea	Jan 2013 (Hiseq2000)
*A. mellifera*	Reproducing	Drone cells	Goroka, Papua New Guinea	Jan 2014 (Hiseq2000)
*A. mellifera*	Reproducing	Drone cells	Goroka, Papua New Guinea	Jan 2014 (Hiseq2000)

^a^SC = San Cristobel, Salomon Islands

^b^Non-reproducing, individual adult females were pooled together expecting to get more RNA for sequencing

### RNA extraction and sequencing

Pools of adult female mites from each sample were ground in liquid nitrogen, and total RNA was extracted using the Invitrogen TRIzol® reagent protocol with one exception; the RNA precipitation step was slightly modified by the addition of 250 μl of RNA precipitation solution (1.2 M NaCl + 0.8 M Sodium citrate dihydrate) mixed with 250 μl of isopropanol to the aqueous phase of the mite homogenate to help precipitate more RNA. Approximately 20 mites per sample were used for extraction, except for the non-reproducing mite sample for which only five non-reproducing mites were available for sequencing and RNA from all 5 mites was pooled. Total RNA per sample was then assessed for quality using a NanoDrop 2000/2000c (Thermosceintific) and submitted to the Purdue University Genomics Core Facility (PGCF) for sequencing. Total RNA was further analyzed for quality and concentration using an Agilent Technologies 2100 Bioanalyzer (Agilent Technologies, Inc. Santa Clara. CA). Seven out of nine cDNA libraries were prepared and barcoded by PGCF using the TruSeq™ RNA sample preparation kit (Illumina, Inc. San Diego, CA). These libraries were prepared and sequenced at two different time points (April 2012 and January 2013) using the Illumina HiScanSQ (100 b paired-end reads, two lanes) and Hiseq2000 (100 b paired-end reads, 4 lanes) systems, respectively (Table 1). The remaining two cDNA libraries were prepared and sequenced using a Hiseq2000 (100 b paired-end reads, one lane) at the Biomolecular Resource Facility (BRF), Canberra, Australia (February 2014). Raw sequence reads from all 9 samples were then analyzed together.

### Read pre-processing

Viral, bacterial, mitochondrial, and ribosomal RNA sequences were removed from the raw reads using the DeconSeq v 0.4.3 software [26], in order to focus on transcripts originating from the nuclear genome. “Contaminant” libraries were created by downloading all sequences in each corresponding category from the NCBI database. The viral library contained a total of 30,300 sequences of complete viral genomes. The bacterial library contained 2,451,824 complete genomic sequences. The mitochondrial library contained the complete sequence of *V. destructor* mitochondrial genome and the ribosomal RNA library contained 28,314 sequences including *V. destructor* 18S and 28S ribosomal RNA sequences. Remaining decontaminated reads were checked for duplicates, and adapters removed using in-house Perl scripts. Sequence quality was assessed using FastQC (v 0.10.0, http://www.bioinformatics.babraham.ac.uk/projects/fastqc/) and quality trimming was performed using Trimmomatic v 0.30 [27], trimmomatic SE -phred33 ILLUMINACLIP:adapters.fa:2:35:15 LEADING:7 TRAILING:7 SLIDINGWINDOW:4:13 MINLEN:30 (Table 1). Because the reads were independently quality trimmed, some reads were unpaired after quality trimming and application of the minimum length cut off (30 bases).

### Transcriptome assembly

A hybrid transcriptome assembly was created using all the paired and unpaired reads from the nine sequenced samples (BioProject: PRJNA321056, SRA: SRP075576). In order to create this hybrid assembly, two different transcriptome assemblies were created first, using the software Trinity (trinity_beta_Jan28_2014) [28], and then these two assemblies were merged using the Program to Assemble Spliced Alignments (PASA; v pasa_r20130907) [29]. A *de novo* transcriptome assembly was created using Trinity default parameters (kmer length = 25, min_contig_length = 200 nucleotides). An independent genome guided assembly was created using (Trinity --genome_guided_max_intron 11000); using as a reference a draft of the genome of *V. destructor* (Jay Evans personal communication, December 2013). For this assembly, an *in silico* normalization of the full data set was performed using Trinity (normalize_by_kmer_coverage.pl --max_cov 50) in order to minimize the CPU running time. Finally, we created the hybrid transcriptome assembly by merging the *de novo* and genome-guided assemblies using PASA (default parameters). The numbers of sequences per assembly reported in this paper differ slightly from the transcriptome assemblies deposited to DDBJ/EMBL/GenBank (*de novo* transcriptome accession: GETM00000000, genome guided assembly accession: GETO00000000 and trinity/PASA hybrid transcriptome accession: GETP00000000) due to transcripts removed during NCBI curation process.

### Description of a gene according to Trinity

Since a high quality genome assembly is available for neither *V. jacobsoni* nor *V. destructor*, it is important to carefully define what we mean by a gene. For *de novo* assemblies, Trinity reports many predicted transcripts, which are generated by combining all the splice junctions observed in the data; some of these predicted isoforms are not observed experimentally. In the first stage of Trinity reads are clustered according to their sequence overlap into components. Components are further divided into subcomponents and predicted isoforms. The concept of a gene most closely matches the component level as determined by BLAST comparisons (data not shown), therefore we performed differential expression (DE) analysis at the component level. In the genome guided assembly, genes are grouped according to their alignment to the reference genome. Each group then is independently assembled using the *de novo* Trinity assembly process. In the PASA hybrid assembly, *de novo* assemblies that do not match to the genome guided assembly are reported with their original Trinity component IDs, and *de novo* assemblies that match the genome guided assembly are merged with the genome guided assembly and combined into PASA assembly clusters (genes) based on exon overlap. Therefore in our analyses a gene indicates a Trinity component, or a PASA assembly cluster.

### Assessing quality of the assembly

To assess the quality of the final assembled transcripts, all RNASeq cleaned reads were aligned back to the hybrid assembly using Bowtie2 [30] and overall mapping statistics were examined. In addition, to evaluate the completeness of the transcriptome assembly, the CEGMA (Core Eukaryotic Genes Mapping Approach), [31] software was applied to identify the presence of a core protein set consisting of 248 highly conserved proteins that are found in a wide range of eukaryotes.

### Assembly annotation

A comprehensive automated functional annotation of the final hybrid assembled transcripts was performed using Trinotate (Transcriptome Functional Annotation and Analysis, [32]. Trinotate makes use of a number of comprehensive annotation databases for functional annotation including homology searches of sequence data (NCBI-BLAST), protein domain identification (HMMER/PFAM), protein signal prediction (siganlP/tmHMM), and comparison to other databases (EMBL UniProt/Swissprot eggNOG/GO pathways). To annotate the assembled transcripts, we also conducted a complete Blastx similarity search against the UniProt/Swissprot protein database, predicted peptides (20,486 sequences/descriptions as of August 22, 2014) of the deer tick *Ixodes scapularis,* with an *E*-value cutoff of ≤ 1e-06, and predicted peptides (11,767 sequences/descriptions as of November 6, 2014) of the mite *Metaseiulus occidentalis*.

### Differential expression analysis

Quantification of the assembled transcripts was performed using standalone RSEM [33] which evaluates transcript abundances by mapping the RNAseq reads to the assembled transcriptome using the aligner tool Bowtie2. Only the reads from eight samples were mapped back to the assembled transcriptome, five samples were from pooled mites that were reproducing in the *A. mellifera* host from PNG, and three samples of pooled mites were reproducing in the *A. cerana* host from the Solomon Islands. Briefly, RSEM calculates posterior mean estimates, 95% credibility intervals, and maximum likelihood abundance estimates or expected counts (EC) for genes and predicted transcripts.

### Identifying consistently differentially expressed mite genes CDEG

Expected counts per gene per sample were combined into a count matrix, and this matrix was used as input for all downstream expression analyses. These analyses were performed using three different R packages EBSeq, EdgeR and DESeq2 [34–36]. All differentially expressed genes that were common among the three methods, using a False Discovery Rate (FDR) ≤ 0.05, were extracted and used for downstream analyses. We refer to these genes as consistently differentially expressed genes (CDEG).

EdgeR is a Bioconductor-R package used to call differentially expressed genes from read counts obtained from RNA-Seq [36]. EdgeR was used to normalize the EC (obtained from RSEM) for relative expression and effective library size using the Trimmed Mean of *M*-values (TMM) normalization method. Genes with at least 0.18 counts per million (CPM), which corresponds to 14 read counts per gene, in at least three samples were selected for further differential expression analysis. Differentially expressed genes (DEG) with FDR ≤ 0.05 and log fold change (logFC) of two were extracted.

The DESeq2 v. 1.0.19 [34] Bioconductor-R package was also used to call differentially expressed genes. DESeq2 implements a negative binomial based model. Before performing the DE analysis, DESeq2 automatically performs independent filtering of the genes with low counts (weakly expressed) in order to maximize the number of DEG with adjusted *P*-values less than a critical value of 0.1. For the differential expression analysis, genewise dispersions were estimated and DEG with FDR ≤ 0.05 and a logFC of two were extracted.

EBSeq v 1.4.0 is a Bioconductor-R package that uses empirical Bayesian methods to identify differentially expressed genes [35]. EBSeq estimates the posterior probability of being differential expressed (PPDE). A list of DE genes with a FDR controlled at α was extracted using a PPDE value greater than 1–α, where α was set to 0.05. By default EBSEq removes transcripts for which fewer than 75% of the samples have greater than 10 counts.

### Heatmap and gene clustering

To generate heatmaps and gene clustering, we used the R packages EdgeR and heatmap3. FPKM (Fragments Per Kilobase per Million) values obtained from RSEM for each of the CDEG were normalized and log_2_ transformed prior to gene clustering. CDEG were clustered according to their patterns of differential expression (correlation distance) using complete linkage clustering. In addition, we used the Trinity script (define_clusters_by_cutting_tree.pl –-Ktree 5). Plots of the expression patterns for the CDEG were generated using a modified version of the Trinity script (plot_expression_patterns.pl).

### GO enrichment analyses of the CDEG

The 37,661 genes that passed the CPM cutoff used in EdgeR were further analyzed using Blast2GO [37] to assign gene ontology (GO) terms to each transcript. Predicted transcripts/genes were compared to the NCBI non-redundant database using Blastx. We retained the best hit for each gene with an *E*-value ≤ 1e-06. Blast2GO and GO enrichment analysis was performed for each CDEG cluster, using the target genes (37,661) as the reference set, and the individual gene clusters as test sets. A *P*-value cutoff of 0.1 was used for GO enrichment test.

## Results

### *Varroa jacobsoni* assembled transcriptome

*V. jacobsoni* mite samples were collected from two different honey bee hosts, *A. cerana* and *A. mellifera,* and from two different geographic locations, the Solomon Islands and PNG, respectively (Table 1). A total of nine RNAseq libraries were constructed and sequenced using two Illumina sequencing platforms (Table 1), yielding a total of 2.18 billion paired-end reads (1.09 billion paired-end fragments) see Table 2.

**Table 2:** Sequencing reads and mapping summary

Sample-ID	Raw reads	Contaminants	Adapters	Trimmed reads	Clean reads	Mapped reads
Ac-reproductive	154,854,698	31,372,885	5,868,855	2,684,238	114,928,720	109,400,649 (95.19%)
Ac-reproductive	376,336,622	96,168,948	3,863,069	5,785,505	270,519,100	259,373,713 (95.88%)
Ac-reproductive	460,610,232	167,624,944	3,759,060	6,705,901	282,520,327	269,524,392 (95.40%)
Am-not reproductive	10,427,368	2,019,087	2,294,687	535,882	5,577,712	5,306,077 (95.13%)
Am-reproductive	146,287,844	27,746,078	871,943	1,942,313	115,727,510	111,179,419 (96.07%)
Am-reproductive	203,052,598	30,479,539	1,337,684	3,330,200	167,905,175	161,793,427 (96.36%)
Am-reproductive	209,363,152	44,563,144	1,102,128	2,502,475	161,195,405	153,861,014 (95.45%)
Am-reproductive	264,092,696	91,851,166	4,377,311	2,797,800	165,066,419	157,324,804 (95.31%)
Am-reproductive	303,036,016	79,679,461	2,046,179	4,996,895	216,313,481	206,579,374 (95.50%)
Undetermined^a^	56,563,734	20,373,131	1,259,132	4,851,428	30,080,043	28,693,353 (95.39%)
Total reads	2,184,624,960	591,878,383	26,780,048	36,132,637	1,529,833,892	1,463,036,222

^a^Reads where the barcode could not be decoded. The order of the sample-ID is the same as in Fig. 1

**Fig. 1:**
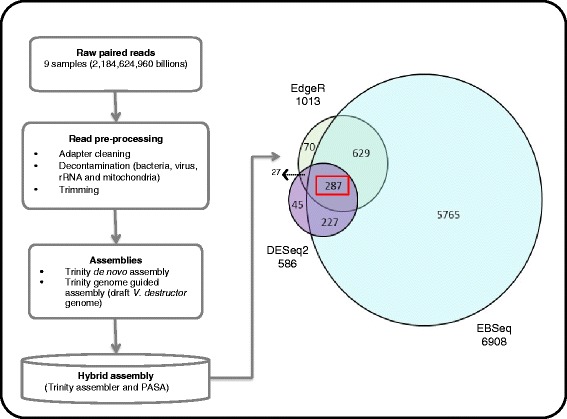
Transcriptome assembly and differentially expressed genes. Pipeline steps followed to build the assembly and expression profiles using 3 different R packages (EBSeq, EdgeR and DESeq2). Flow chart shows the steps implemented from raw reads to the selection of the final hybrid assembly and the selection of the consistently differentially expressed mite genes (CDEG)

After pre-processing of the raw reads, a total of 592 million contaminant (viral, bacterial, mitochondrial and rRNA) reads (27%) and 26.7 million (1.2%) reads with adapters were removed from the raw data set. Furthermore, a total of 36.1 million (1.6%) reads with low quality were removed, leaving a total of 1.53 billion reads (70%) that were used for the transcriptome assemblies (Table 2). Three different transcriptome assemblies were created using Trinity/PASA as described in methods, see Fig. 1 for a detailed workflow). The final hybrid assembly produced a total of 319,231 putative transcripts and 223,620 putative genes (N50 = 3549 bp). The numbers reported here are before transcriptome assembly was deposited to DDBJ/EMBL/GenBank (this transcriptome shotgun assembly project has been deposited under the accession GETP00000000, this version here is the first version, GETM01000000). It is well known that the *de novo* transcriptome assemblers predict many more transcripts than are actually present due to the difficulty in predicting complete isoforms from short reads [38, 39]. In the quality analysis below, all predicted transcripts were used – the results are therefore reported in terms of the coverage of the reference sequences. In the subsequent gene expression analysis, all predicted transcript isoforms of each gene are combined, so the overprediction of isoforms is not an issue.

### Assessing the quality of the assembly

The details of the three assemblies as described in methods are shown in Table 3. The quality and completeness of our hybrid *V. jacobsoni* transcriptome assembly was assessed in four different ways: using CEGMA [31], by comparison with predicted gene sequences of the tick *I. scapularis* and the predatory mite *M. occidentalis* [40], and by aligning the cleaned reads back to the hybrid assembly.

**Table 3:** Description of assemblies of *Varroa jacobsoni*

Assembly type	Putative transcripts	Putative genes	N50
Trinity de novo	374,530^a^	252,445	3406 bp
Trinity genome-guided	428,912^a^	155,121	6266 bp
Hybrid (trinity/PASA)	319,231^a^	223,620	3549 bp

^a^The numbers reported here are before transcriptome assemblies were deposited to DDBJ/EMBL/GenBank (accessions GETM00000000, GETO00000000, GETP00000000, respectively)

Analysis of our hybrid assembly against the CEGMA protein set identified 246 out of 248 core proteins (99.2%) as complete (defined as > 70% alignment length versus the core protein) see Table 4. Furthermore, an average of about three *V. jacobsoni* assembled transcripts (perhaps representing 3 splice variants) aligned with each core protein, with 221 of those detected having more than 1 alignment (Table 5). We compared the hybrid assembly against the UniProt/Swissprot database using Blastx (hits with *E*-value ≤ 1e-06) and identified 4957 proteins represented by nearly full-length transcripts, having > 80% alignment coverage, and 8372 proteins having > 50% alignment coverage. In addition, we compared the hybrid assembly against the *I. scapularis* and the *M. occidentalis* predicted peptides database, using Blastx (*E*-value ≤ 1e-06). From the search against the *I. scapularis* database we found 3957 proteins that are represented by nearly full-length transcripts, having > 80% alignment coverage, and 5925 proteins having > 50% alignment coverage, which represents ~29% of the deer tick predicted peptides. Furthermore, from the search against the *M. occidentalis* database we found 5362 proteins that are represented by nearly full-length transcripts, having > 80% alignment coverage, and 7063 proteins having > 50% alignment coverage, which represents 60% of the total predatory mite predicted peptides. Cleaned reads for each sample were mapped back to the hybrid reference assembly using Bowtie2. Overall 95.6% of reads aligned to the reference indicating that almost all reads are represented in the assembly (Table 2). In summary, the *V. jacobsoni* transcriptome assembly contains a good representation of the core eukaryotic genes (CEGs), and a large portion of the reference peptides from related genera are represented in the mite transcriptome as substantially complete transcripts, together suggesting that the transcriptome described here is relatively complete.

**Table 4:** Completeness of the *V. jacobsoni* transcriptome based on 248 CEGs

	# Prots^a^	% Completeness^b^	# Total^c^	Average^d^	% Ortho^e^
Complete^f^	246	99.19	807	3.28	89.84
Group 1	66	100.00	230	3.48	90.91
Group 2	56	100.00	196	3.5	91.07
Group 3	60	98.36	182	3.03	85.00
Group 4	64	98.46	199	3.11	92.19
Partial^g^	248	100.00	967	3.9	98.39
Group 1	66	100.00	271	4.11	96.97
Group 2	56	100.00	229	4.09	100.00
Group 3	61	100.00	221	3.62	98.36
Group 4	65	100.00	246	3.78	98.46

These results are based on the set of genes selected by Genis Parra

^a^Prots = number of 248 ultra-conserved CEGs present in genome, ^b^%Completeness = percentage of 248 ultra-conserved CEGs present, ^c^Total = total number of CEGs present including putative orthologs, ^d^Average = average number of orthologs per CEG, ^e^%Ortho = percentage of detected CEGS that have more than 1 ortholog, ^f^Complete = refers to those predicted proteins in the set of 248 CEGs that when aligned to the HMM for the KOG for that protein-family, give an alignment length that is 70% of the protein length, ^g^Partial = If a protein is not complete, but if it still exceeds a pre-computed minimum alignment score

**Table 5:** Most specific GO terms related to mite genes that are down-regulated in the *A. mellifera* host, cluster 1

GO-ID	Term	Category	*P*-Value	Am-down seq. count^a^	Ref seq. count^b^
GO:0060237	regulation of fungal-type cell wall organization	P	0.000611	1	1
GO:0000978	RNA polymerase II core promoter proximal region sequence-specific DNA binding	F	0.000102	2	56
GO:0000987	core promoter proximal region sequence-specific DNA binding	F	0.000181	2	75
GO:0001159	core promoter proximal region DNA binding	F	0.000195	2	78
GO:0048546	digestive tract morphogenesis	P	0.000440	2	118
GO:0003705	RNA polymerase II distal enhancer sequence-specific DNA binding transcription factor activity	F	0.000549	2	132
GO:0000977	RNA polymerase II regulatory region sequence-specific DNA binding	F	0.000697	2	149
GO:0001012	RNA polymerase II regulatory region DNA binding	F	0.000782	2	158
GO:0048565	digestive tract development	P	0.001380	2	211
GO:0055123	digestive system development	P	0.001610	2	228

Fisher’s exact test showing enriched GO terms in mite genes that are down-regulated in *A. mellifera* host (cluster 1). For a complete list and gene ID see (Additional file 5: Table S5). ^a^23 genes in test set ^b^number of times the GO was identified in reference set of 37,661 genes

### Annotation of the assembly at transcript and gene levels

The hybrid transcriptome assembly of *V. jacobsoni* was used to query entries described in the UniProt/Swissprot protein database, using Blastx (*E*-value ≤1e-06). Only the most significant hit for each predicted transcript was retained. At the transcript level we found that 51,025 (~16%) out of 319,231 predicted transcripts have a match to a protein sequence, and 2870 (~6%) of those matches have a sequence identity ≥ 90%. These numbers seem very reasonable; alternatively spliced predicted transcripts are expected to have more than one match.

At the gene level we found 24,128 out of 223,620 putative genes have a match to a protein sequence and 2413 (10%) of them have a sequence identity ≥ 90%. Furthermore, when we compared the hybrid transcriptome assembly of *V. jacobsoni* against the *I. scapularis* database, which contains 20,486 unique predicted peptides, we found that 21,333 (~9%) of the *V. jacobsoni* genes, had a significant hit to a protein sequence in the deer tick database (E ≤ 1e-06). On the other hand, these 21,333 blast hits covered only 7629 (37.3%) of the predicted peptides of the deer tick. When we compared it against the *M. occidentalis* predicted peptides (11,767 unique sequences), we found that only 23,779 (~11%) of the *V. jacobsoni* genes had a significant hit to a protein sequence in the predatory mite (E ≤ 1e-06). However, these 23,779 blast hits covered 8388 (~71%) of the predicted peptides of the predatory mite. These results are expected if we consider that these two mites species are taxonomically classified under the same Mesostigmata order.

### Differential expression analysis of mites reproducing on *A. cerana* and mites reproducing on *A. mellifera*

Quantification of the assembled transcripts was performed using standalone RSEM. Transcript abundances were evaluated by mapping the RNA-Seq cleaned reads to the assembled hybrid transcriptome using the aligner tool Bowtie2. The transcript abundance distribution looks very similar for all samples, indicating the data are suitable for differential expression analysis (see Additional file 1; Figure S1, histograms distribution).

To identify differentially expressed genes we used three different R packages as described in methods. Only eight of the nine mite samples were included in differential expression analysis; We chose not to include the Am-non-reproductive mite sample, because we did not have a biological replicate, and the reproductive status of the adult females was not the same as in the other eight samples. In addition, the number of reads obtained during sequencing was lower than for the rest of samples (Table 2).

After removing genes with low counts using EdgeR, we evaluated 37,661 genes for differential expression. We focused on those genes that were differentially expressed according to all three methods (EBSeq, EdgeR and DESeq2) in order to have a conservative set of consistently differentially expressed genes (CDEG) Fig. 1. EdgeR identified 1013 differentially expressed genes (FDR < 0.05 and absolute logFC ≥ 2; see Additional file 2; Table S1). In addition, using DESeq2 and EBSeq we identified a total of 586 and 6809 DEG (FDR < 0.05), respectively (see Additional file 3; Table S2 and Table S3). A total of 287 CDEG common to all three methods were extracted and further analyzed (Fig. 1). Out of the 287 CDEG, we found a total of 23 down-regulated genes and 264 up-regulated genes in the mites reproducing in the *A. mellifera* host as compared to mites on the *A. cerana* host.

**Fig. 2:**
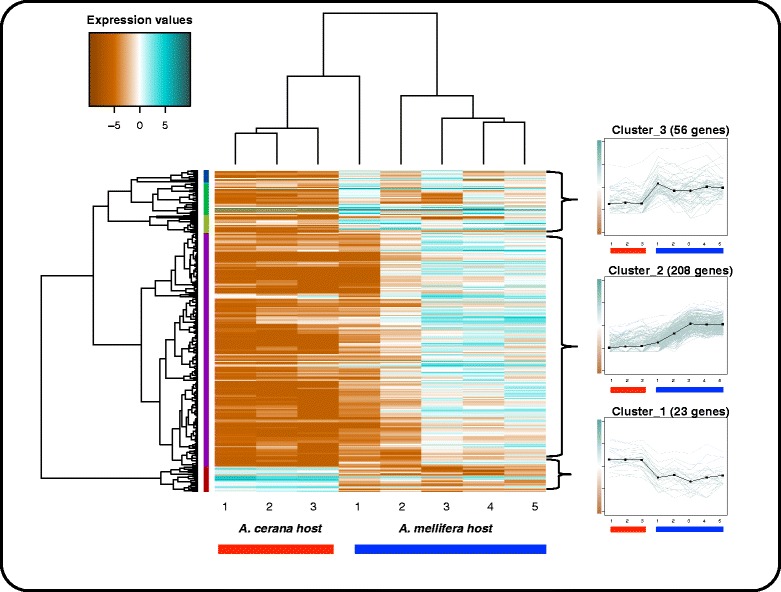
Heatmap and gene clusters of CDEMG genes for *V. jacobsoni* mites. Heatmap of expression values (log2 transformed normalized FPKM) of the CDEMG adult female *V. jacobsoni* mites reproducing in *A. cerana* and *A. mellifera*. Orange and turquoise blue indicate higher and lower expression values, respectively. Red and blue tick bars indicate the *A. cerana* host and *A. mellifera* host respectively

We grouped the genes according to expression pattern using correlation distances between genes and complete linkage clustering. Originally five gene clusters were generated, however, based on visual inspection of the pattern of expression we manually clustered the CDEG into three groups (Fig. 2). Cluster one contains 23 CDEG that were down-regulated in mites reproducing on the *A. mellifera* host. Cluster two contains 208 CDEG; differences in expression of genes in this cluster are not clearly correlated with the host on which the mite was reproducing. Cluster three contains 56 genes that were up-regulated in all mites reproducing on the *A. mellifera* host. Although generally up-regulated in the *A. mellifera* host, genes in clusters two and three showed clear differences in their pattern of expression across samples (Fig. 2). For example, in cluster two we observed that four samples (three *A. cerana* samples plus one *A. mellifera* sample) showed consistent low expression patterns across all 208 mite genes, while the other four *A. mellifera* samples showed higher expression patterns. However, cluster 3 showed more consistent up-regulation of mite genes across all *A. mellifera* samples.

### GO terms assignment and Enrichment analysis

We used Blast2GO to assign GO terms to the 287 CDEG, and to test whether certain biological functions or GO terms are more frequently observed in the gene clusters, we used the Fisher exact test in Blast2GO to compare the GO terms of the CDEG in each of the gene clusters versus those in the complete transcriptome (reference set of 37,661 genes) each gene was represented by the highest Blastx hit and a *P*-value 0.1 was used for the Fisher exact test, see (Additional file 4; Table S4).

### Cluster 1 CDEG down-regulated in *A. mellifera*

We found 23 CDEG that were specifically down-regulated in mites reproducing on the *A. mellifera* host. However, GO terms could be assigned for only two of these genes. The GO terms associated with these genes are related to digestive tract development and transcription factors (Table 5). For the full report of all the GO terms and the 36 unique GO-ID associated with cluster 1 genes, see Additional file 5: Table S5. Furthermore, only 5 of the 23 genes had a significant Blastx similarity hit (*E*-value ≤ 1e-06) to the *M. occidentalis* predicted peptides (see Additional file 6: Table S6) and only 3 out the 23 genes had significant Blastx similarity hits (*E*-value ≤ 1e-06) to the UniProt/Swissprot database. It makes sense that mites feeding in a suboptimal host will show differences in digestive tract development and this might be mediated by transcriptional regulation.

### Cluster 2 and 3 CDEG up-regulated in *A. mellifera*

We found 208 CDEG up-regulated in *A. mellifera* in cluster 2. However, only eight out of these 208 genes had an associated GO term. The top 10 enriched GO terms associated with these genes are primarily involved in either oxidative metabolism and stress (mitochondrial respiratory chain complex, oxidoreductase complex) or in development and reproduction (developmental process involved in reproduction, germ cell development, establishment of endothelial barrier, cis-Golgi network, post-embryonic organ development, cellular process involved in reproduction; Table 6). For a full report see Additional file 7: Table S7. Only 80 out of the 208 genes had a significant Blastx similarity hits (*E*-value ≤ 1e-06) to the *M. occidentalis* predicted peptides (Additional file 6: Table S8) and only 88 out the 208 genes had significant Blastx similarity hits (*E*-value ≤ 1e-06) to the UniProt/Swissprot database. We found 56 CDEG up-regulated in *A. mellifera* contained in cluster 3. However, only two out of these 56 genes had a GO term associated with them. Visual inspection of the GO terms associated with these genes are related to either apoptosis (Bcl-2 family protein complex and B cell apoptotic process, BH-domain binding), or the following terms: epoxide hydrolase activity, leukotriene metabolic process, ether hydrolase activity and Type I pneumocyte differentiation (Table 6). For a full report see Additional file 8: Table S9. Furthermore, only 15 out of the 56 genes had a significant Blastx similarity hits (*E*-value ≤ 1e-06) to the *M. occidentalis* predicted peptides (Additional file 6: Table S10) and only 12 out the 56 genes had significant Blastx similarity hits (*E*-value ≤ 1e-06) to the UniProt/Swissprot database.

**Table 6:** Most specific GO in mite genes that are up-regulated in the *A. mellifera* host, cluster 2 and 3

GO-ID	Term	Category	*P*-Value	Am-Up seq. count^a^	Ref seq. count^b^
Cluster 2 (208 CDEG)					
GO:0005746	mitochondrial respiratory chain	C	0.000198	4	61
GO:0016272	prefoldin complex	C	0.000182	2	6
GO:0003006	developmental process involved in reproduction	P	0.000016	24	1332
GO:0010029	regulation of seed germination	P	0.000233	2	7
GO:0007281	germ cell development	P	0.000119	14	665
GO:1990204	oxidoreductase complex	C	0.000855	5	85
GO:0061028	establishment of endothelial barrier	P	0.000476	3	16
GO:0005801	cis-Golgi network	C	0.000169	3	26
GO:0048569	post-embryonic organ development	P	0.000254	11	489
GO:0048610	cellular process involved in reproduction	P	0.000267	18	1065
Cluster 3 (56 CDEG)					
GO:0097136	Bcl-2 family protein complex	C	0.000016	1	1
GO:0051400	BH domain binding	F	0.000057	1	6
GO:0001783	B cell apoptotic process	P	0.000089	1	10
GO:0004301	epoxide hydrolase activity	F	0.000033	1	3
GO:0004463	leukotriene-A4 hydrolase activity	F	0.000041	1	4
GO:0060509	Type I pneumocyte differentiation	P	0.000049	1	5
GO:0019370	leukotriene biosynthetic process	P	0.000057	1	6
GO:0016803	ether hydrolase activity	F	0.000073	1	8
GO:0016801	hydrolase activity, acting on ether bonds	F	0.000097	1	11
GO:0006691	leukotriene metabolic process	P	0.000138	1	16

Fisher’s exact test showing enriched GO terms in mite genes that are up-regulated in *A. mellifera* host (cluster 2 and 3). For a complete list and gene ID see (Additional file 7: Table S7; Additional file 8: Table S9). ^a^208 and 56 genes in each test set, respectively. ^b^number of times the GO was identified in reference set of 37,661 genes

## Discussion

In this study we look at the relative expression of mite genes with respect to the host on which they were found reproducing. Because, there is no external standard, is it impossible to estimate absolute expression levels. For brevity, we refer to genes that have lower relative expression in mites reproducing on *A. mellifera* compared to mites reproducing in *A. cerana*, as down-regulated.

### Functions of consistently differentially expressed genes

An analysis of the functions of the consistently differentially expressed mite genes (CDEG) that were differentially expressed (DE) between *V. jacbosoni* mites that differed in their ability to parasitize European honey bees revealed several trends. Only 23 transcripts were down-regulated in mites reproducing on *A. mellifera*. These included genes coding Proteins with RNAII polymerase promoter-region specific DNA-binding activity, as well as genes involved in digestive tract development. These results suggest that some transcription factors are down-regulated on this host and are possibly involved in the host-parasite interaction. Genes involved in digestive tract development are also interesting because feeding on the host may influence their expression. However, the obvious and surprising trend overall is that 91% of the DE transcripts were more highly expressed in mites using *A. mellifera* as a host.

The two largest classes of genes that were more highly expressed in mites growing on *A. mellifera* hosts contained nuclear encoded mitochondrial genes and genes involved in metabolic regulation and apoptosis. The overall Up-regulation of genes involved in some primary metabolism as described above may be a result of stress induced in the mites. These results suggest that these mites may have been exposed to more stress than they would have been on the optimal host *A. cerana*. Included in this broad category were genes encoding 8 mitochondrial proteins, a heatshock protein and a conserved NAD+ sensing histone deacetylase,SIRT6, that regulates glucose homeostasis in mammals [41]. Other CDEG that have roles in cellular primary metabolism were observed including 6-phosphofructokinase, a coordinator of glucose metabolism and cell cycle, phospholipase A2 activating protein, involved in calcium/CaMKII signaling, and a phosphodiesterase 8A homolog, a regulator of cyclic AMP levels [42–44]. Up-regulation of genes involved in primary metabolism may be a result of stress induced in the mites living on an atypical host. However, *V. jacobsoni* reproduction is not restricted to drone brood on *A. mellifera*, but is also found reproducing in the worker brood, which might indicate that this mite is rapidly adapting to live and reproduce in their new host.

Stress induced by a number of treatments in *Drosophila* results in increased expression of mitochondrial and heat shock genes [45]. Interestingly, genes putatively involved in reproductive development and growth were also more highly expressed in mites reproducing on *A. mellifera*. For example, a transcript with highly significant alignment to *Drosophila* Src64 was over expressed. This gene encodes a tyrosine kinase that is required for Drosophila oogenesis, and affects insulin signaling through interactions with the transcription factor dFOXO [46, 47].

Our samples come from populations that differ in their ability to reproduce on *A. mellifera,* but not only were they exposed to different host colony environments, they were also geographically separated, perhaps confounding our differential expression analyses. However, other analyses indicate that our samples of mites parasitizing *A. mellifera* are likely derived from the same source population as the mites we collected parasitizing *A. cerana* [25], and colony environments are buffered from external climactic conditions. These differential expression analyses provide a valuable resource for future studies into the mechanisms involved in this singular host shift to European honey bees. Discovering why mated mites fail to lay eggs upon entering brood cells of different honey bee host species is critical to our understanding of this devastating pest species, and for predicting the ability of *Varroa* mites to successfully make a host switch to *A. mellifera*.

## Conclusions

Some genes are differentially expressed in the mites reproducing on *A. mellifera* and *A. cerana*, with more variation in gene expression among mites that reproduce on *A. mellifera*. A small set of genes showed reduced expression in mites on the *A. mellifera* host, including putative transcription factors and digestive tract developmental genes. The vast majority of differentially expressed genes were up-regulated in this host. This gene set showed enrichment for genes associated with mitochondrial respiratory function and apoptosis, suggesting that mites on this host may be experiencing higher stress, and may be less optimally adapted to parasitize it. The restricted ability of *V. jacobsoni* mites to successfully reproduce on the *A. mellifera* host, to which they are incompletely adapted, may be related to this stress. Some genes involved in reproduction and oogenesis were also differentially expressed, which should be further studied in regard to this host shift.

## Additional files

Additional file 1: Figure S1. Distribution of normalized FPKM. Histograms of the normalized FPKM values in the different samples showing the distribution of these values about the median. (PDF 211 kb)
Additional file 2: Table S1.
*Varroa jacobsoni*, 1013 differentially expressed genes (FDR < 0.05 and absolute logFC ≥ 2) using EdgeR. (XLSX 145 kb)
Additional file 3: Table S2.
*Varroa jacobsoni*, 586 differentially expressed genes (FDR < 0.05) using DESeq2. **Table S3.** *Varroa jacobsoni*, 6908 differentially expressed genes (FDR < 0.05) using EBSeq. (XLSX 604 kb)
Additional file 4: Tables S4. Selected target genes (37,661) used as a reference set for Fisher’s exact test. (XLSX 2572 kb)
Additional file 5: Table S5. GO terms significantly over-represented in mite genes that are down-regulated in the *A. mellifera* host, cluster 1 (23 genes). (XLS 43 kb)
Additional file 6: Table S6. Genes that had a significant Blastx similarity hit (*e*-value ≤ 1e-06) to the *M. occidentali*s predicted peptideds (cluster 1). **Table S8.** Genes that had a significant Blastx similarity hit (*e*-value ≤ 1e-06) to the *M. occidentali*s predicted peptideds (cluster 2). **Table S10.** Genes that had a significant Blastx similarity hit (*e*-value ≤ 1e-06) to the *M. occidentali*s predicted peptideds (cluster 3). (XLSX 45 kb)
Additional file 7: Table S7. GO terms significantly over-represented in mite genes that are down-regulated in the *A. mellifera* host, cluster 2 (208 genes). (XLSX 13 kb)
Additional file 8: Table S9. GO terms significantly over-represented in mite genes that are down-regulated in the *A. mellifera* host, cluster 3 (56 genes). (XLS 29 kb)

## Abbreviations

CPM: Counts per million
EC: Expected counts
FPKM: Fragments per kilobase per million reads
GO: Gene ontology
RNA-Seq: RNA sequencing
