# Supplementary figures and images for: Differential gene expression in Varroa jacobsoni mites following a host shift to European honey bees (Apis mellifera)
Source: BMC Genomics. 2016 Nov 16;17:926. doi: 10.1186/s12864-016-3130-3 (PMC5112721; doi:10.1186/s12864-016-3130-3)

**Figure S1. Distribution of normalized FPKM**

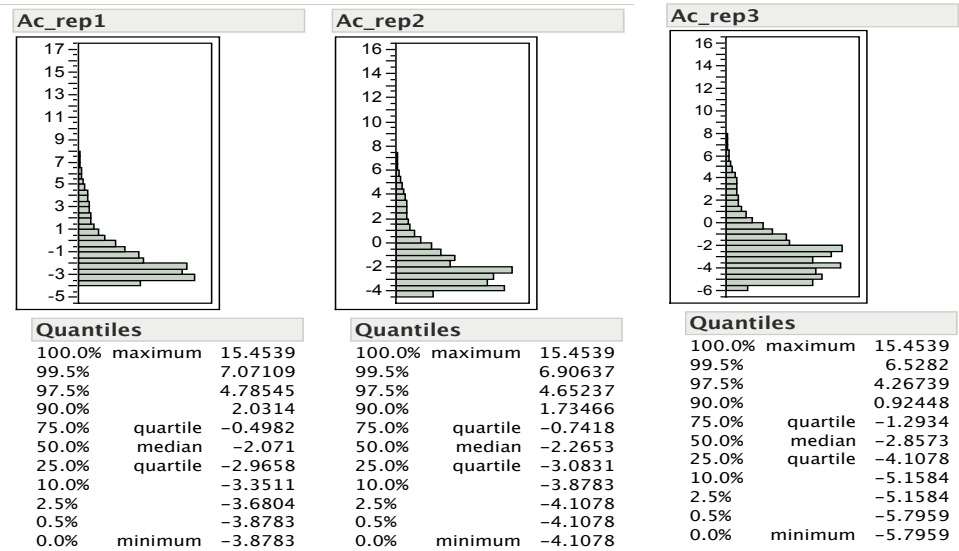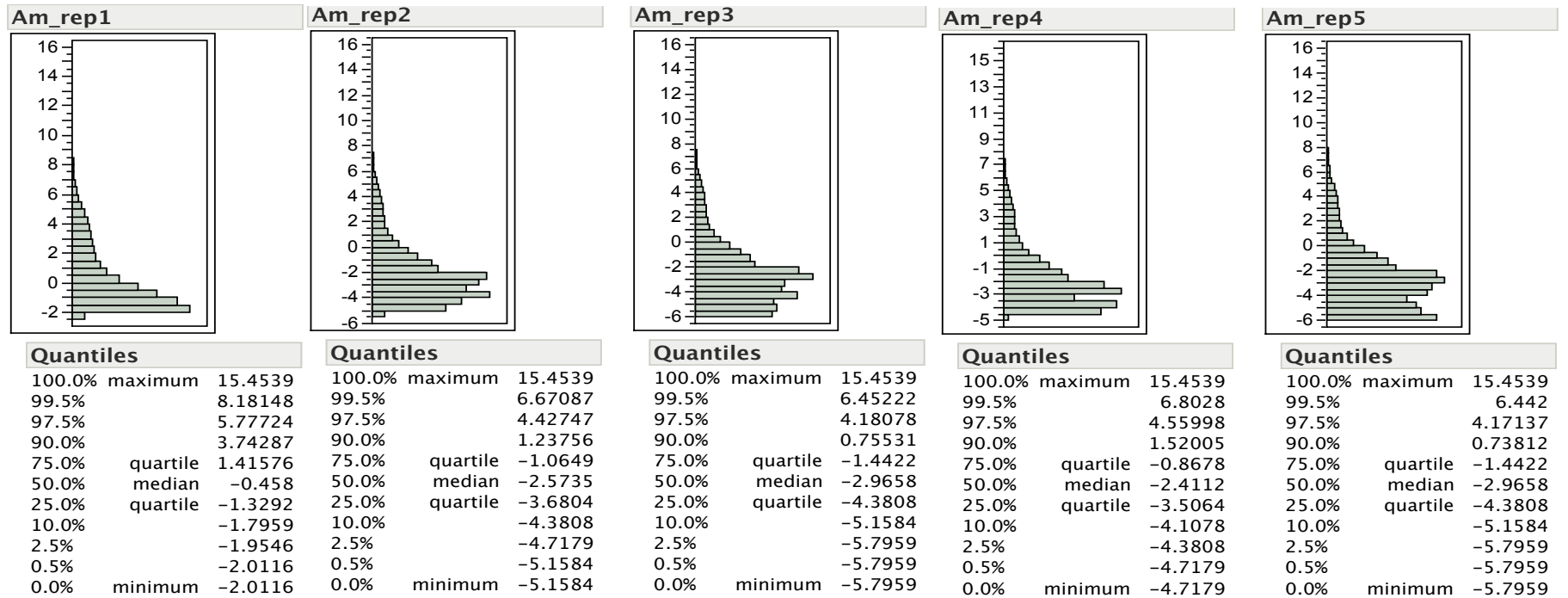

Supplement: Additional file 1: Figure S1. — Distribution of normalized FPKM. Histograms of the normalized FPKM values in the different samples showing the distribution of these values about the median. (PDF 211 kb) [file 12864_2016_3130_MOESM1_ESM.pdf]
